# A Histological Assessment Tool for Breast Implant Capsules Validated in 480 Patients with and Without Capsular Contracture

**DOI:** 10.1007/s00266-024-04128-5

**Published:** 2024-06-07

**Authors:** Andreas Larsen, Adam Mandrup Timmermann, Mikela Kring, Tim Kongsmark Weltz, Mathias Ørholt, Peter Vester-Glowinski, Jens Jørgen Elberg, Jesper Trillingsgaard, Louise Vennegaard Mielke, Lisbet Rosenkrantz Hölmich, Tine Engberg Damsgaard, Anne Roslind, Mikkel Herly

**Affiliations:** 1 Department of Plastic Surgery and Burns Treatment, Copenhagen University Hospital, Rigshospitalet, Blegdamsvej 9, 2100 Copenhagen, Denmark; 2 Amalieklinikken, Copenhagen, Denmark; 3 AK Nygart, Copenhagen, Denmark; 4 Aleris, Copenhagen, Denmark; 5 Department of Plastic and Reconstructive Surgery, Herlev and Gentofte, Copenhagen University Hospital, Copenhagen, Denmark; 6 Department of Clinical Medicine, University of Copenhagen, Copenhagen, Denmark; 7 Department of Plastic and Reconstructive Surgery, Odense and Little Belt Hospital, Odense University Hospital, Vejle, Denmark; 8 Department of Regional Health Research, University of Southern Denmark, Odense, Denmark; 9 Department of Pathology, Herlev and Gentofte, Copenhagen University Hospital, Copenhagen, Denmark; 10 Department of Immunology and Microbiology, University of Copenhagen, Copenhagen, Denmark

**Keywords:** Breast implants, Capsular contracture, Fibrosis, Foreign-body reaction, Histology, Inflammation

## Abstract

**Background:**

Understanding the impact of breast implants on the histological response in the surrounding fibrous capsule is important; however, consensus is lacking on how to analyze implant capsules histologically. We aimed to develop a standardized histological assessment tool to be used in research potentially improving diagnostic accuracy and treatment strategies for capsular contracture.

**Methods:**

Biopsies of breast implant capsules from 480 patients who had undergone breast augmentation or reconstruction were collected and stained with hematoxylin and eosin. Initially, biopsies from 100 patients were analyzed to select histological parameters demonstrating the highest relevance and reproducibility. Then, biopsies from the remaining 380 patients were used to determine intra- and interobserver agreements of two blinded observers and agreement with a pathologist. Finally, we tested the association between the parameters and capsular contracture.

**Results:**

The histological assessment tool included ten parameters assessing the inflammatory, fibrotic, and foreign-body reaction to breast implants, each graded on two-, three-, or four-point scales. Intra- and interobserver agreements were almost perfect (0.83 and 0.80), and agreement with the pathologist was substantial (0.67). Four parameters were significantly correlated with capsular contracture, namely chronic inflammation with lymphocyte infiltration (*p* < 0.01), thickness of the collagen layer (*p* < 0.0001), fiber organization (*p* < 0.01), and calcification (*p* < 0.001).

**Conclusions:**

This is the first validated histological assessment tool for breast implant capsules. The validated tool not only advances our understanding of capsular contracture but also sets a new standard for histological evaluation in breast implant research and clinical diagnostics.

**No Level Assigned:**

This journal requires that authors assign a level of evidence to each submission to which Evidence-Based Medicine rankings are applicable. This excludes Review Articles, Book Reviews, and manuscripts that concern Basic Science, Animal Studies, Cadaver Studies, and Experimental Studies. For a full description of these Evidence-Based Medicine ratings, please refer to the Table of Contents or the online Instructions to Authors www.springer.com/00266.

**Supplementary Information:**

The online version contains supplementary material available at 10.1007/s00266-024-04128-5.

## Introduction

Capsular contracture is one of the most common severe complications of breast implant surgery. Within 10 years after surgery, capsular contracture affects 3.6–19% [1–5] of women after breast augmentation and 3.3–25% [1, 3–6] of women after implant-based breast reconstruction. The severity of capsular contracture is most often clinically evaluated using the Baker Classification System, which consists of a four-point scale based on the firmness, distortion of the breast, and pain [7, 8]. Despite the importance of capsular contracture, its pathogenesis is still unclear [9, 10]. Previous studies suggest that capsular contracture is caused by a low-grade inflammatory response in the tissue around the breast implant, which results in excessive fibrosis [11–13]. In rare cases, the immunological reaction may potentially be the cause of breast implant-associated anaplastic large cell lymphoma (BIA-ALCL); however, our knowledge of these immunological processes is still very limited [14, 15].

Several studies have investigated the histological characteristics of breast implant capsules. However, previous studies are limited by descriptive histological approaches in small, heterogeneous patient populations, focusing on individual parameters such as capsule thickness or synovial-like metaplasia [16]. Combining parameters previously reported in the literature on breast implant capsule histology into a standardized and reproducible assessment tool, will create a common method between studies, facilitate effective comparison of results, and improve our ability to build on previous knowledge. Such a tool may help us understand why some but not all women develop capsular contracture, and it may be of use in developing new preventive strategies.

To address this gap in the literature, this study introduces a validated semiquantitative assessment tool aimed for a systematic evaluation of key histological parameters associated with the foreign-body reaction to breast implants. The assessment tool is developed for hematoxylin and eosin (H&E)-stained biopsies from breast implant capsules and is intended to be used for the evaluation of capsules from all types of breast implants. Furthermore, we aimed to show the applicability of the assessment tool by analyzing differences between patients with and without capsular contracture.

## Materials and Methods

### Patients and Sample Material

The histological samples were obtained from the Copenhagen Breast Implant (COBI) Biobank of tissue biopsies excised from breast implant capsules. Patients were eligible for inclusion to the biobank if they had previously undergone implant-based breast reconstruction or breast augmentation and were scheduled for exchange or removal of their breast implants. Patients were excluded in case of ongoing breast infection, pregnancy, or current breastfeeding. Patients were included from October 2019 to August 2022 from two plastic surgery departments (Rigshospitalet, Department of Plastic Surgery and Burns Treatment and Herlev and Gentofte Hospital, Department of Plastic Surgery) and three private hospitals (Amalieklinikken, AK Nygart, and Aleris). Patient demographics and implant characteristics were obtained from the patients’ medical chart. The Baker grade was assessed preoperatively by the surgeon prior to sample acquisition. Patient and implant characteristics are described in Table 1. The biobank consisted of samples from 400 patients who had their implants removed after breast augmentation and 80 patients who had their implants removed after an implant-based breast reconstruction. Biopsies of approximately 1 cm^2^ were excised from the anterior-inferior part of the fibrous capsule adjacent to the inframammary fold, fixated in a 4% formaldehyde solution and embedded in paraffin. Sections of 3–4 µm were cut and stained with hematoxylin and eosin (H&E). All sections were digitalized using a whole slide scanner and analyzed using NDP.viewer2 (Hamamatsu Photonics).

**Table 1:** Patient and implant characteristics

	Breast augmentation	Breast reconstruction
Patients, n	400	80
Breasts, n	400	80
Median age, yr (IQR)	45.9 (38.1–54.2)	56.9 (49.7–67.2)
Median BMI, kg/m^2^ (IQR)	22.3 (20.5–24.6)	24.5 (22.1–26.9)
*Smoking, pt (%)*		
Yes	98 (24)	4 (5)
No	302 (76)	76 (95)
*ASA score, pt (%)*		
I	258 (65)	23 (29)
II	123 (31)	47 (59)
III	8 (2)	8 (10)
Unknown	10 (2.5)	2 (2.5)
*Indication for revisional surgery, pt (%)*		
Capsular contracture	200 (50)	33 (41)
Obs. rupture	64 (16)	10 (13)
Obs. BIA-ALCL	10 (2.5)	2 (2.5)
Implant age	7 (1.8)	0 (0.0)
Cosmetic reason	119 (30)	35 (44)
*Baker grade, breast (%)*		
I	147 (37)	23 (29)
II	53 (13)	24 (30)
III	110 (28)	23 (29)
IV	90 (23)	10 (13)
*Debut of capsular contracture, breast (%)*		
Under 1 year	47 (24)	7 (30)
Over 1 year	145 (73)	16 (70)
Unknown	8 (4.0)	0 (0.0)
Median time of implantation, yr, breast (IQR)	14.0 (8.0–18.0)	5.0 (2.2–11.8)
*Implant plane, breast (%)*		
Subpectoral	336 (84)	67 (84)
Prepectoral	63 (16)	2 (2.5)
Unknown	1 (0.3)	11 (14)
*Implant brand, breast (%)*		
Mentor	157 (39)	51 (64)
EuroSilicone	92 (23)	1 (1.3)
Nagor	6 (1.5)	0 (0.0)
Allergan/McGhan	40 (10)	14 (18)
CUI	19 (4.8)	0 (0.0)
Motiva	11 (2.8)	1 (1.3)
Silimed	4 (1.0)	0 (0.0)
Polytech	2 (0.5)	2 (2.5)
Other	12 (3.0)	0 (0.0)
Unknown	56 (14)	11 (14)
*Implant rupture, breast (%)*		
Yes	105 (26)	10 (13)
No	295 (74)	63 (79)
*Implant texture, breast (%)*		
Smooth	36 (9.0)	1 (1.3)
Textured	364 (91)	79 (99)
*Previous implant exchange, breast (%)*		
Yes	67 (17)	17 (21)
No	333 (83)	63 (79)
*Radiotherapy, breast (%)*		
Yes	–	11 (14)
No	–	69 (86)
*Acellular dermal matrix, breast (%)*		
Yes	–	22 (28)
No	–	58 (72)

Yr = year, IQR = interquartile range, BMI = body mass index, pt = patients, ASA score = American Society of Anesthesiology Physical Status Classification Score

### Design of the Semiquantitative Assessment Tool

**Fig. 1:**
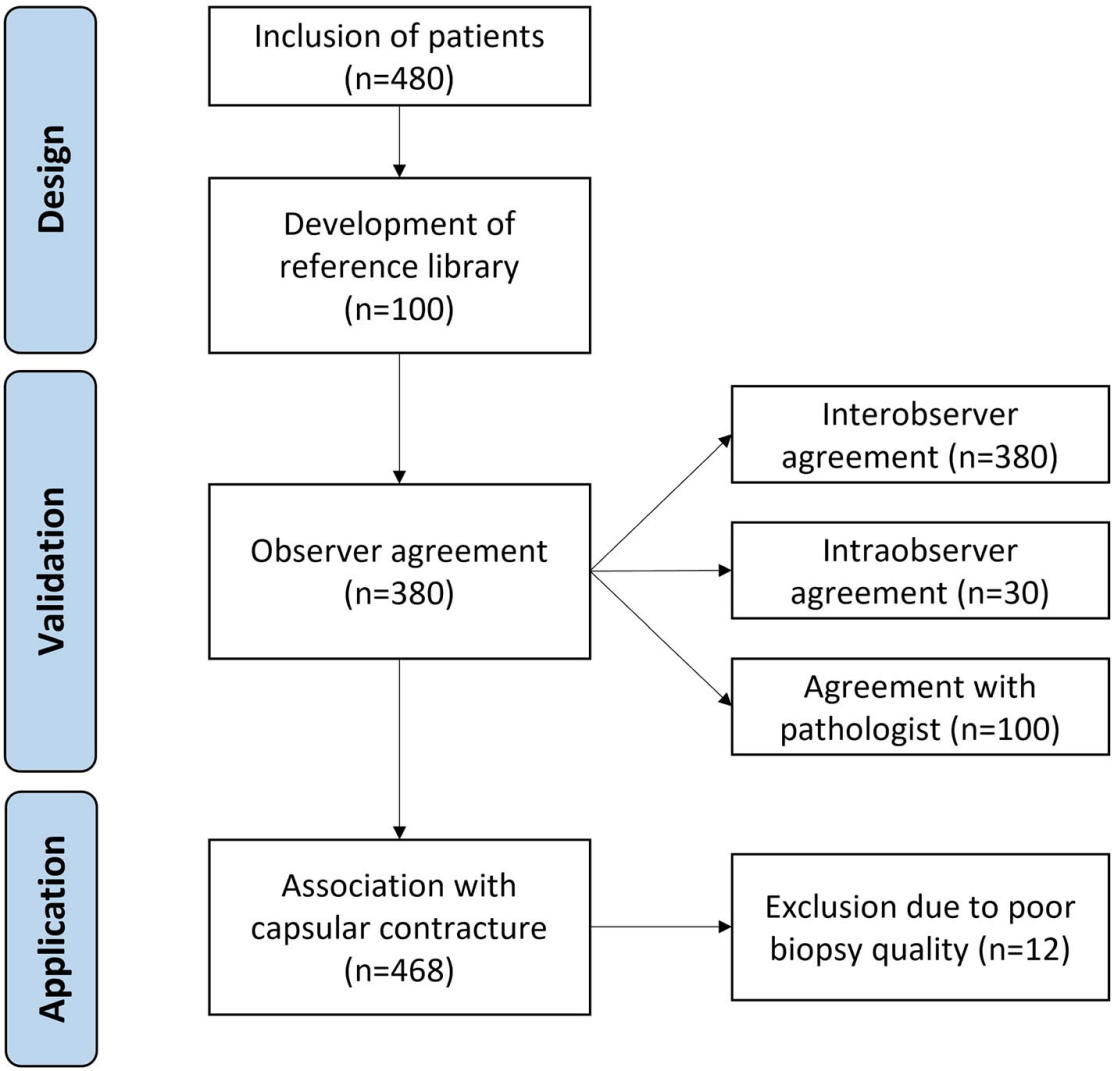
Flow diagram of the study design

The semiquantitative assessment tool was designed and validated according to principles for valid histologic scoring in research [17]. Potential parameters for the histological assessment tool were chosen by the authors based on a systematic review of histological studies of breast implant capsules [16]. Each selected parameter was transferred to a binary scale or an ordinal scale from absent to severe to assess the prevalence and abundance of each parameter in the biopsies. The reproducibility and applicability of each potential parameter were tested by two observers on breast implant biopsies from 100 randomly selected women from the biobank. Disagreement between observers was discussed with a breast pathologist until a consensus was reached. Parameters were chosen based on prevalence in biopsies excluding those with low incidence or low reproducibility after initial testing. The ordinal scales were modified to ensure that each scale point was used in the sample. To ensure consistency and accuracy in histological assessment and to prevent diagnostic drift, we developed a comprehensive reference library with more than 60 histological images. Each parameter included in the assessment tool is visualized with histological images that illustrate the full range of possible scores. The reference library, available in Electronic Supplementary Material 1, provides detailed visual guides, information on the location and morphology of each parameter, and specific instructions on how to assign a semiquantitative score to each. In the next phase, the assessment tool was validated in terms of observer reproducibility in the samples from the remaining 380 women in the biobank. Two observers scored all 380 samples. In addition, 30 of these samples were scored twice to calculate intraobserver agreement. The biopsies from 100 randomly selected patients were scored by the breast pathologist to assess the agreement between the pathologist and the observers. The results from the histological scorings were used to assess the association between each parameter and the patient’s clinical severity of capsular contracture using the Baker grade. A flowchart of the study design is presented in Fig. 1.

### Ethical Considerations

Oral and written informed consents were obtained from all patients prior to inclusion in the study in compliance with the Declaration of Helsinki. The study was approved by the Regional Ethics Committee (H-20015276) and the Danish Data Protection Agency (P-2020-176 and P-2020-177).

### Statistics

Agreement between and within observers was calculated with linear weighted Kappa scores to fit the ordinal scales of the histological parameters [18]. Interobserver agreement compared the scores of each histological parameter between two observers, and intraobserver agreement compared the duplicated scores of each histological parameter. Kappa scores were evaluated as no (0.01–0.20), fair (0.21–0.40), moderate (0.41–0.60), substantial (0.61–0.80), and almost perfect (0.81–1.00) agreement in accordance with the criteria set Cohen et al. [19]. The association between the Baker grade (Baker I/II vs. Baker III/IV) and the histological parameter was estimated with a multivariable logistic regression with ridge penalization and robust covariance estimation to adjust for clustering of observers in accordance with the CLUDA guidelines [20]. All associations were reported as odds ratios with 95% CIs, and p values were evaluated at an alpha level of 5%. All statistics and plots were performed in R, version 4.2.0.

## Results

### Selection of Parameters

We identified 16 potential parameters for the semiquantitative assessment tool. Four parameters were related to fibrosis, including fibroblast and myofibroblast quantification, thickness of the dense collagen layer and organization of the collagen fibers. Four parameters were related to inflammation, including acute inflammation (neutrophil granulocytes), chronic inflammation (lymphocytes), vascularization and calcification. Five potential parameters were related to a foreign-body reaction, including synovial-like metaplasia, free silicone vacuoles, intracellular located silicone in macrophages (foam cells), multinucleated giant cells and granulomatous inflammation (silicone granulomas). Furthermore, the total number of layers in the capsule, activity in the stromal layer and neuromas were identified as potential parameters for the assessment tool.

Based on the initial scoring of the 100 randomly selected biopsies, five parameters were excluded, including acute inflammation, granulomatous inflammation, number of layers, neuromas and histomorphometric grading of synovial-like metaplasia [21] due to low prevalence, low applicability, or low reproducibility. Ten parameters were included in the final assessment tool, each with its own two-, three- or four-point scale. An overview of the parameters in the assessment tool can be seen in Table 2. The thickness of the collagen layer was divided into four intervals based on the median and interquartile range (IQR) to enable assignment of a semiquantitative score. Reliable morphological distinction between fibroblasts, myofibroblasts and resident macrophages is challenging in H&E-stained sections. Consequently, we chose to combine these into one parameter that evaluates the number of resident cells in the collagen layer. Furthermore, we combined free silicone vacuoles and foam cells into one parameter for silicone infiltration.

**Table 2:** The histological semiquantitative assessment tool for breast implant capsules

	1	2	3	4
*Fibrosis*				
Thickness of collagen layer	<400 µm	400-600 µm	600-800 µm	>800 µm
Fiber organization	Highly disorganized	Moderately disorganized	Highly organized	–
Resident cells in the collagen layer	Few fibroblasts and macrophages	Several fibroblasts and macrophages	Many fibroblasts and macrophages	–
*Inflammation*				
Lymphocyte infiltration	Few single lymphocytes	Several single lymphocytes or few lymphocyte aggregates	Severe infiltration of lymphocytes with several follicular aggregates	–
Calcification	Absent	Present	–	–
Vascularization	Low	High	–	–
*Foreign-body-reaction*				
Synovial-like metaplasia	Absent	1–2 cell layers	3–4 cell layers	> 5 cell layers
Silicone deposition	Absent or nearly absent	Few silicone vacuoles and/or foam cells	Several silicone vacuoles and/or foam cells	Abundant silicone vacuoles and/or foam cells
Giant cells	Absent	Few giant cells	Several giant cells	–
Activity in stromal layer	Absent stromal layer	Present with low cellularity	Present with high cellularity	–

### Reliability and Reproducibility of the Histological Assessment Tool

The intra- and interobserver agreements were 0.85 (95% CI 0.85–0.85) and 0.80 (95% CI 0.80–0.80), respectively, which indicate almost perfect agreement. Six biopsies (1.5%) from patients with breast augmentation and six biopsies (7.5%) from patients with breast reconstruction were excluded due to poor quality of the histological slides (for instance, insufficient representation of the full capsule, tissue folding or sectioning artifacts). Examples of poor-quality biopsies have been included in the reference library (ESM1). The agreement between the observers and the breast pathologist was 0.67 (95% CI 0.67–0.67), indicating substantial agreement.

### Histological Features of the Breast Implant Capsule

**Fig. 2:**
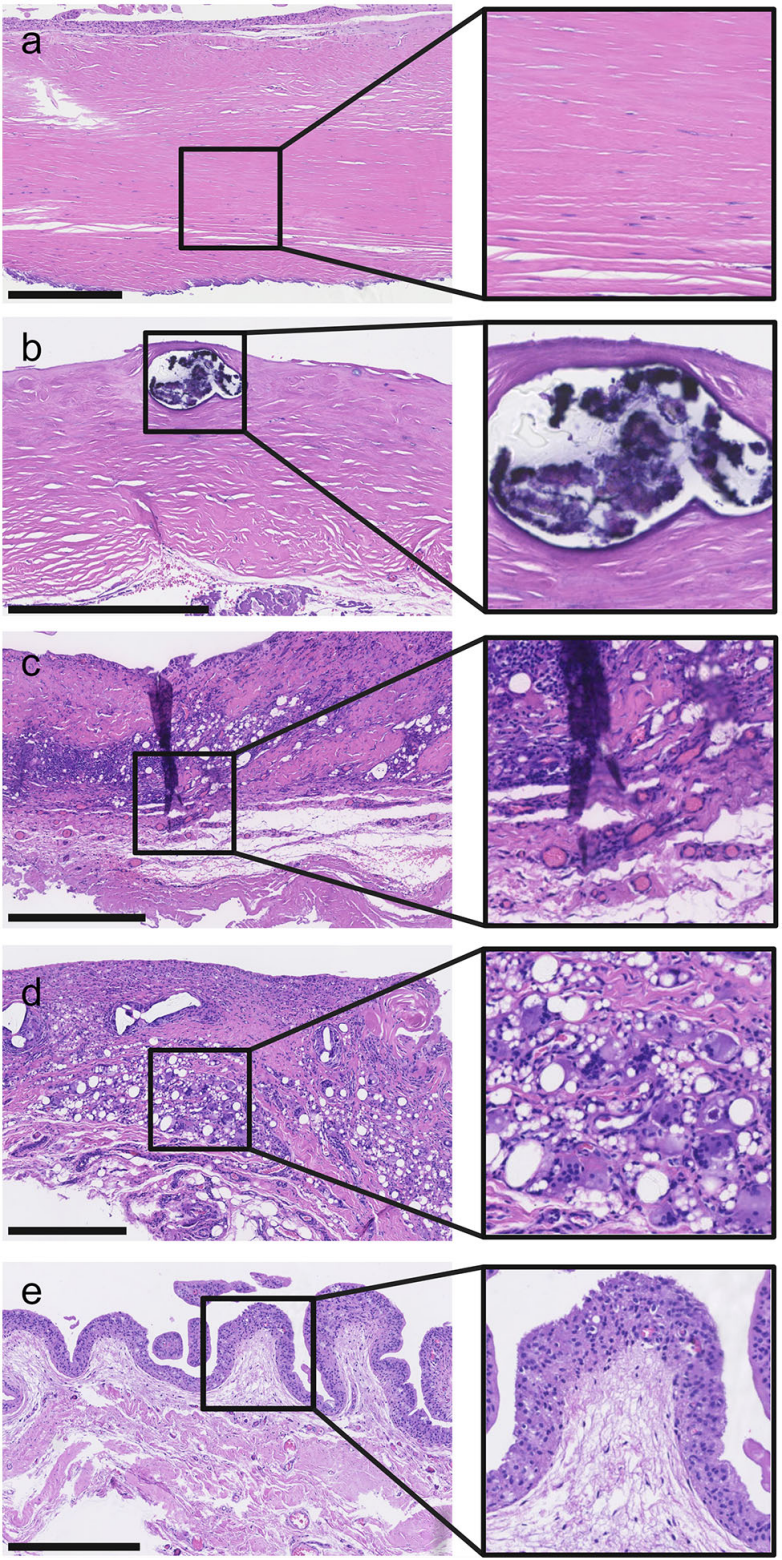
Examples of the histological appearance of breast implant capsules. All scale bars are 500 µm; **a** capsule with highly organized fibers (3 points) running parallel to the implant surface and fibroblast nuclei elongated in the direction of traction, **b** capsule with a low cellularity of resident cells (1 points) and the presence of calcification (2 points), **c** capsule with severe chronic inflammatory with lymphocyte infiltration (3 points) and high vascularization (2 points), **d** capsule with abundant silicone vacuoles and foam cells (4 points) and several multinucleated giant cells (3 points), and **e** capsule with synovial-like metaplasia of 3–4 cell layers (3 points) and the presence of a stromal layer with low cellularity (2 points)

When investigating the fibrotic changes, we found that the collagen layer generally constituted the majority of the implant capsule biopsies and varied greatly in thickness and fiber organization. The thickness of the collagen layer ranged from 72 µm to over 3000 µm. We found that 19% of the samples were highly organized with fibers running parallel to the implant surface and fibroblast nuclei elongated in the direction of traction and parallel to the fibers (Fig. 2a). In contrast, 14% of the samples were highly disorganized with multidirectional bundles of fibers and crosscut, rounder fibroblast nuclei. The cellularity of the resident cells in the collagen layer also varied greatly, with 24% of the samples containing many fibrocytes, fibroblasts, myofibroblasts and macrophages, whereas 12% of the samples had a low cellularity with an almost acellular appearance (Fig. 2a, b).

When investigating the inflammatory response, we found chronic inflammation with infiltration of lymphocytes to be present in 12% of the samples (Fig. 2c). Tissue calcification was generally rare and only seen in 6% of the samples (Fig. 2b). Furthermore, we found a low degree of vascularization in most of the biopsies (85%); however, as expected, a high degree of vascularization was often seen in biopsies with severe lymphocyte infiltration (Fig. 2c).

**Fig. 3:**
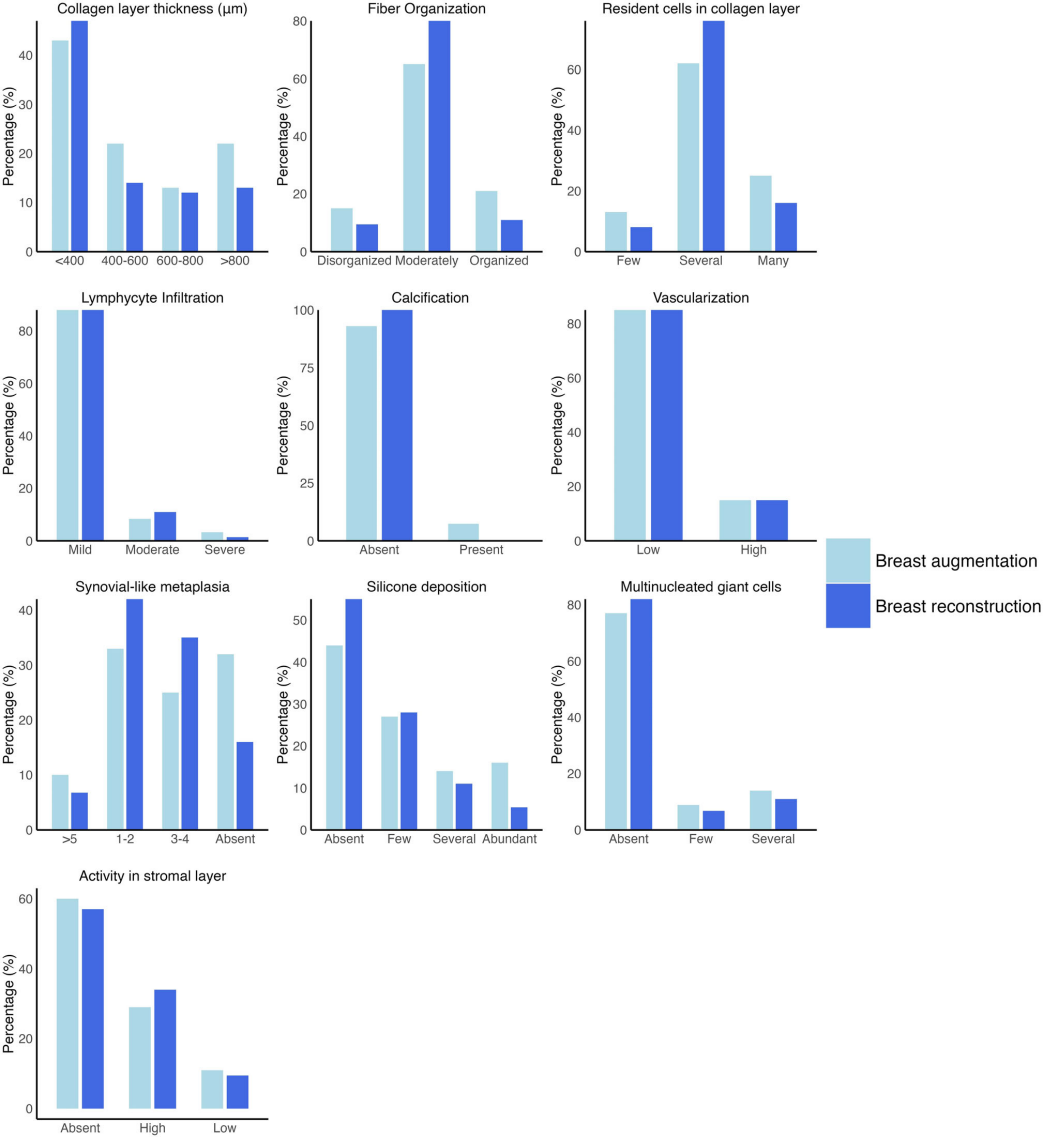
Distribution of histological scores between biopsies from patients with breast augmentation and breast reconstruction

Silicone was observed extracellularly as small round to irregular translucent droplets of amorphous refractile material as well as intracellularly when phagocytized by histiocytes, giving them a foam cell appearance (Fig. 2d). Silicone was identified in over half of the included samples (54%). Multinucleated giant cells, which are known to be part of a foreign-body reaction, were seen in 22% of the samples and were closely related to the presence of silicone vacuoles and foam cells (Fig. 2d). A layer of synovial-like metaplasia was found adjacent to the implant surface in 70% of the samples with a varying thickness from 1 to > 5 cell layers (Fig. 2e) but was absent in 30% of the samples (Fig. 2b). Moreover, we found a layer of stromal tissue to be present between the synovial-like metaplasia and the layer of dense collagen fibers or between bundles of densely packed collagen fibers in 42% of the samples (Fig. 2e). See Fig. 3 for the distributions of histological scores between biopsies from patients with breast augmentation and breast reconstruction.

### Associations between the Histological Parameters and Capsular Contracture

**Fig. 4:**
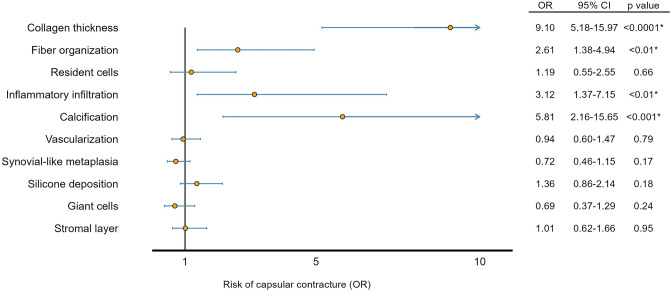
The association between each histological parameter and the clinical degree of capsular contracture estimated with a multivariable logistic regression

To demonstrate the applicability of the semiquantitative assessment tool, the association between the histological scores and Baker grade was analyzed in 468 patients. We found that four fibrotic and inflammatory parameters were significantly associated with capsular contracture. The thickness of the collagen layer had the strongest association with capsular contracture: 40% of the capsules from breasts with capsular contracture (Baker III/IV) had the highest score with a thickness above 600 µm compared with only 13% of the capsules from breasts without capsular contracture (Baker I/II) (*p* < 0.0001). Moreover, the collagen fibers were significantly more organized with fibers running parallel to the implant surface in capsules from breasts with capsular contracture than in capsules from breasts without capsular contracture (28% vs. 14%, *p* < 0.01). Infiltration of lymphocytes in the fibrous capsule was also significantly more present in capsules from breasts with capsular contracture than in capsules from breasts without capsular contracture (17% vs. 6%, *p* < 0.01). Furthermore, the presence of calcification was also significantly associated with capsular contracture (14% vs. 0.7%, *p* < 0.001). The remaining parameters were not significantly associated with capsular contracture (Fig. 4).

## Discussion

To the best of our knowledge, this is the first validated histological semiquantitative assessment tool that can be used to evaluate the immunological and fibrotic response in breast implant capsules. The assessment tool comprises 10 semiquantitative parameters that are highly reliable in terms of intra- and interobserver agreements and with substantial agreement between the observers and a breast pathologist. Histological evaluations in a clinical setting should always be performed by a trained pathologist. However, we have shown that histological assessments with this new tool can also be performed by non-pathologists trained in using the assessment tool under the supervision of a pathologist when using the tool for research purposes. To improve the applicability and usability of the assessment tool, we created a large histological reference index with histological examples of each parameter and thorough instructions on how to score each parameter. We found no acute inflammation or neuromas and only one true silicone granuloma, and consequently, these parameters were discarded from the semiquantitative assessment tool. Our semiquantitative assessment tool provides an objective and standardized measure for characterizing the histology of breast implant capsules. It was validated in 480 patients after breast augmentation or breast reconstruction. The provided distributions of the individual parameters may serve as a strong reference point for future studies. The semiquantitative assessment tool was developed for H&E-stained sections and does not require special immunohistochemical staining, which makes it cost-efficient to include in future studies. The semiquantitative assessment tool can be applied to other important research questions, such as the impact of implant texture, radiotherapy and biological or artificial meshes. Therefore, we recommend that future studies include all 10 parameters and not just those significantly associated with capsular contracture, as we might find other important patterns when including all the visible histological characteristics of breast implant capsules.

As an example of the applicability of the semiquantitative assessment tool, we analyzed the differences between patients with and without capsular contracture and found two fibrotic and two inflammatory parameters significantly associated with capsular contracture. The correlation between capsule thickness and capsular contracture is well established in the literature.[12, 22–25] In a study by Siggelkow et al. [12] analyzing 53 biopsies from 43 patients, a significant association of capsule thickness and Baker grade was found, but they did not find an association with inflammation, silicone deposition or calcification. Bui et al. [23] also found capsules from breasts with capsular contracture to be significantly thicker than those from breasts without capsular contracture based on the analysis of 49 samples from 41 patients. In line with our findings, they found capsules from breasts with capsular contracture to have a significantly higher degree of fiber organization than capsules from breasts without capsular contracture based on vector analysis. Most previous studies investigating the inflammatory reaction to breast implants have either been based on qualitative analysis or have not included statistical tests [26–28]. In a study by Kamel et al. [29] analyzing 93 biopsies, they did, however, find a significant association between the Baker grade and CD3-positive lymphocytes, but they did not find a correlation with calcification. In histological studies of capsular contracture, calcification is described as the end stage of chronic inflammation and the fibrotic reaction [30, 31]. In a study by Peters et al., [32] calcification was clinically present in 16% of breast implant capsules and was significantly more frequent in first-generation implants [33] than in second- and third-generation implants. In our study, we primarily included biopsies from breasts with textured fourth- or fifth-generation implants and found that calcification was present histologically in 14% of capsules from breasts with capsule contracture and 0.7% of capsules from breasts without capsular contracture. This may be the explanation why previous studies with smaller sample sizes have not been able to find a significant correlation between calcification and capsular contracture [12, 26, 27, 29]. Previous studies have described a loss of cellularity in the fibrous capsule when the time of implantation increases [31]. Myofibroblasts are hypothesized to undergo apoptosis in the later stages of capsular contracture development, similar to what has been observed in wound healing and scar formation [23]. The role of fibroblasts and myofibroblasts in capsular contracture and how the cellularity change over time may be elucidated using specific immunohistochemical stains that improve the distinction between the two cell types, which is not possible in H&E-stained sections [34–37]. Previous studies have found an inverse relationship between the presence of synovial-like metaplasia and capsular contracture and have hypothesized that it plays an important protective role against capsular contracture [22, 38]. Raso et al. [39] hypothesized that the protection is caused by the production of lubricating factors such as proteoglycans, whereas De Bakker et al. [22] hypothesized that synovial-like metaplasia is lost due to the increasing amount of tissue and, thereby, loss of nutrition. Other potential reasons could be that the layer is torn off during implant removal due to a strong adhesion between the implant surface and the capsule, which has been described as a “Velcro effect” [16], or that the layer is lost during sample preparation. The results from this study showed no significant association between the absence of synovial-like metaplasia and capsular contracture. Silicone leakage is believed to trigger capsular contracture by inducing an inflammatory foreign-body response [40, 41]. However, we did not find the semiquantitative amount of silicone deposition or the presence of multinucleated giant cells in the capsule to be independently associated with capsular contracture. This could be due to a potential underestimation, as silicone is known to be lost during tissue preparation. Future studies performing a quantitative analysis of silicone leakage adjusted for rupture status may have the sensitivity to find a possible correlation between the amount of silicone in the capsule and capsular contracture [42, 43].

### Future Research Directions

An important perspective for future studies will be to investigate how variables such as implant surface, radiotherapy, and the use of surgical meshes influence the inflammatory-, fibrotic- and foreign-body response in patients with breast implants using the histological assessment tool. Moreover, we believe that the assessment tool would be a valuable addition to clinical studies in need of an objective evaluation of capsular contracture which does not require additional patient visits compared with other techniques for confirming a capsular contracture diagnosis such as MRI or ultrasound [44]. Furthermore, to increase the applicability in clinical practice, another perspective for future studies would be to develop predictive models for the development of capsular contracture by correlating early changes in the breast implant capsule before disease onset to later development of capsular contracture. Future studies should also explore how patient-reported outcomes correlate with parameters of the histological assessment tool. By correlation measures from the BREAST-Q questionnaire with parameters such as chronic inflammation or silicone leakage, we will gain a deeper understanding of how patient-reported outcomes are correlated with histological changes in the breast implant capsule.

## Limitations

The study has some limitations that should be addressed. Our study population predominantly consisted of patients with textured implants (> 90%), which may limit the applicability of our semiquantitative assessment tool for smooth implants which could potentially affect the generalizability of our assessment tool. Future studies are encouraged to verify the applicability in patients with smooth implants to ensure broader applicability. The parameters of the semiquantitative assessment tool are scaled after a population with an even distribution of patients with and without capsular contracture. Therefore, a study that, for example, is concerned with patients with very short implantation times and no capsular contracture will probably find that their patients cluster in one extreme of many of the assessment tool’s parameters. Standardization of the anatomical sample site has improved the comparability between samples; however, this might impose a sampling bias, as potential variability of the histological parameters between different locations of the capsule has not been investigated.

## Conclusion

To our knowledge, this is the largest histological study of the breast implant capsule and the morphological changes from capsular contracture. We have developed and validated a histological semiquantitative assessment tool that is highly reliable in terms of agreement and can be used with a standard H&E stain. Thus, it can provide an accurate and objective assessment of breast implant capsules for future research studies and clinical practice. We strongly encourage the broader research and clinical community to not only use this histological assessment tool but also to actively participate in its ongoing refinement, aiming to improve the understanding of how breast implants impact the inflammatory and fibrotic response in the breast implant capsule and how this is correlated to capsular contracture, thereby improving patient care in breast implant surgery.

## Supplementary Information

Below is the link to the electronic supplementary material.

Electronic Supplementary Material 1. A reference library of histological images illustrating each parameter of the semiquantitative assessment tool with detailed instructions on how to score each parameter.
Supplementary file1 (PDF 4214 kb)

## References

[1] Hammond DC, Canady JW, Love TR et al (2017) Mentor contour profile gel implants: clinical outcomes at 10 years. Plast Reconstr Surg 140:1142–1150. 10.1097/PRS.0000000000003846 29176413 10.1097/PRS.0000000000003846

[2] Calobrace MB, Stevens WG, Capizzi PJ et al (2018) Risk factor analysis for capsular contracture: a 10-year sientra study using round, smooth, and textured implants for breast augmentation. Plast Reconstr Surg 141:20S-28S. 10.1097/PRS.0000000000004351 29595715 10.1097/PRS.0000000000004351

[3] Spear SL, Murphy DK (2014) Natrelle round silicone breast implants: core study results at 10 years. Plast Reconstr Surg 133:1354–1361. 10.1097/PRS.0000000000000021 24867717 10.1097/PRS.0000000000000021 PMC4819531

[4] Cunningham B (2007) The mentor core study on silicone memorygel breast implants. Plast Reconstr Surg 120:19S-32S. 10.1097/01.prs.0000286574.88752.04 18090810 10.1097/01.prs.0000286574.88752.04

[5] Grant Stevens W, Bradley Calobrace M, Alizadeh K et al (2018) Ten-year core study data for sientra’s food and drug administration-approved round and shaped breast implants with cohesive silicone gel. Plast Reconstr Surg 141:7S-19S. 10.1097/PRS.0000000000004350 29595714 10.1097/PRS.0000000000004350

[6] Hvilsom GB, Hölmich LR, Steding-Jessen M et al (2011) Delayed breast implant reconstruction: a 10-year prospective study. J Plast Reconstr Aesthet Surg 64:1466–1474. 10.1016/j.bjps.2011.06.026 21865106 10.1016/j.bjps.2011.06.026

[7] Spear SL, Baker JL, Coffee HH (1995) Classification of capsular contracture after prosthetic breast reconstruction. Plast Reconstr Surg 96:1124. 10.1097/00006534-199510000-00019 7568488

[8] Hu H, Jacombs A, Vickery K et al (2015) Reply: chronic biofilm infection in breast implants is associated with an increased T-cell lymphocytic infiltrate: implications for breast implant-associated lymphoma. Plast Reconstr Surg 135:319–329. 10.1097/PRS.0000000000001243 25383716 10.1097/PRS.0000000000000886

[9] Lu Y, Chen Z, Pan Y, Qi F (2023) Identification of drug compounds for capsular contracture based on text mining and deep learning. Plast Reconstr Surg. 10.1097/PRS.0000000000010350 36862957 10.1097/PRS.0000000000010350

[10] Embrey M, Adams EE, Cunningham B et al (1999) A review of the literature on the etiology of capsular contracture and a pilot study to determine the outcome of capsular contracture interventions. Aesthetic Plast Surg 23:197–206. 10.1007/s002669900268 10384019 10.1007/s002669900268

[11] Doloff JC, Veiseh O, de Mezerville R et al (2021) The surface topography of silicone breast implants mediates the foreign body response in mice, rabbits and humans. Nat Biomed Eng. 10.1038/s41551-021-00739-4 34155355 10.1038/s41551-021-00739-4

[12] Siggelkow W, Faridi A, Spiritus K et al (2003) Histological analysis of silicone breast implant capsules and correlation with capsular contracture. Biomaterials 24:1101–1109. 10.1016/s0142-9612(02)00429-5 12504533 10.1016/s0142-9612(02)00429-5

[13] Basu CB, Leong M, Hicks MJ (2010) Acellular cadaveric dermis decreases the inflammatory response in capsule formation in reconstructive breast surgery. Plast Reconstr Surg 126:1842–1847. 10.1097/PRS.0b013e3181f44674 21124125 10.1097/PRS.0b013e3181f44674

[14] Collett DJ, Rakhorst H, Lennox P et al (2019) Current risk estimate of breast implant-associated anaplastic large cell lymphoma in textured breast implants. Plast Reconstr Surg 143:30S-40S. 10.1097/PRS.0000000000005567 30817554 10.1097/PRS.0000000000005567

[15] Blombery P, Thompson ER, Prince HM (2019) Molecular drivers of breast implant-associated anaplastic large cell lymphoma. Plast Reconstr Surg 143:59S-64S. 10.1097/PRS.0000000000005570 30817557 10.1097/PRS.0000000000005570

[16] Larsen A, Rasmussen LE, Rasmussen LF et al (2021) Histological analyses of capsular contracture and associated risk factors: a systematic review. Aesthetic Plast Surg 45:2714–2728. 10.1007/s00266-021-02473-3 34312696 10.1007/s00266-021-02473-3

[17] Gibson-Corley KN, Olivier AK, Meyerholz DK (2013) Principles for valid histopathologic scoring in research. Vet Pathol 50:1007–1015. 10.1177/0300985813485099 23558974 10.1177/0300985813485099 PMC3795863

[18] Cohen J (1960) A coefficient of agreement for nominal scales. Educ Psychol Meas 20:37–46. 10.1177/001316446002000104

[19] McHugh ML (2012) Interrater reliability: the kappa statistic. Biochem Med (Zagreb) 22:276–282 23092060 PMC3900052

[20] Hemmingsen MN, Nygaard CMT, Kaufmann A et al (2022) How to report data on bilateral procedures and other issues with clustered data: the CLUDA reporting guidelines. Plast Reconstr Surg 150:459–464. 10.1097/PRS.0000000000009293 35687407 10.1097/PRS.0000000000009293

[21] Cheriyan T, Guo L, Orgill DP et al (2012) Lubricin in human breast tissue expander capsules. J Biomed Mater Res B Appl Biomater 100:1961–1969. 10.1002/jbm.b.32763 22865664 10.1002/jbm.b.32763

[22] de Bakker E, van den Broek LJ, Ritt MJPF et al (2018) The histological composition of capsular contracture focussed on the inner layer of the capsule: an intra-donor baker-I versus baker-IV comparison. Aesthetic Plast Surg 42:1485–1491. 10.1007/s00266-018-1211-1 30187083 10.1007/s00266-018-1211-1 PMC6280822

[23] Bui JM, Perry T, Ren CD et al (2015) Histological characterization of human breast implant capsules. Aesthetic Plast Surg 39:306–315. 10.1007/s00266-014-0439-7 25743110 10.1007/s00266-014-0439-7 PMC4434852

[24] Ersek RA, Burroughs JR, Ersek CL, Navarro A (1991) Interrelationship of capsule thickness and breast hardness confirmed by a new measurement method. Plast Reconstruct Surg 87:1069–1073 10.1097/00006534-199106000-00008 2034726

[25] Prantl L, Pöppl N, Horvat N et al (2005) Serologic and histologic findings in patients with capsular contracture after breast augmentation with smooth silicone gel implants: Is serum hyaluronan a potential predictor? Aesthetic Plast Surg 29:510–518. 10.1007/s00266-005-5049-y 16328636 10.1007/s00266-005-5049-y

[26] Prantl L, Schreml S, Fichtner-Feigl S et al (2007) Clinical and morphological conditions in capsular contracture formed around silicone breast implants. Plast Reconstr Surg 120:275–284. 10.1097/01.prs.0000264398.85652.9a 17572576 10.1097/01.prs.0000264398.85652.9a

[27] Luke JL, Kalasinsky VF, Turnicky RP et al (1997) Pathological and biophysical findings associated with silicone breast implants: a study of capsular tissues from 86 cases. Plast Reconstr Surg 100:1558–1565 9385972 10.1097/00006534-199711000-00029

[28] Carpaneda CA (1997) Inflammatory reaction and capsular contracture around smooth silicone implants. Aesthetic Plast Surg 21:110–114. 10.1007/s002669900094 9143426 10.1007/s002669900094

[29] Kamel M, Protzner K, Fornasier V et al (2001) The peri-implant breast capsule: an immunophenotypic study of capsules taken at explantation surgery. J Biomed Mater Res 58:88–96. 10.1002/1097-4636(2001)58:1%3c88::aid-jbm130%3e3.0.co;2-7 11153003 10.1002/1097-4636(2001)58:1<88::aid-jbm130>3.0.co;2-7

[30] Yeoh G, Russell P, Jenkins E (1996) Spectrum of histological changes reactive to prosthetic breast implants: a clinopathological study of 84 patients. Pathology 28:232–235. 10.1080/00313029600169044 8912351 10.1080/00313029600169044

[31] Legrand AP, Marinov G, Pavlov S et al (2005) Degenerative mineralization in the fibrous capsule of silicone breast implants. J Mater Sci Mater Med 16:477–485. 10.1007/s10856-005-6989-0 15875259 10.1007/s10856-005-6989-0

[32] Peters W, Pritzker K, Smith D et al (1998) Capsular calcification associated with silicone breast implants: incidence, determinants, and characterization. Ann Plast Surg 41:348–360. 10.1097/00000637-199810000-00002 9788214 10.1097/00000637-199810000-00002

[33] Bradley Calobrace M, Caprizi PJ (2014) The biology and evolution of cohesive gel and shaped implants. Plast Reconstr Surg 134:6–11 10.1097/PRS.0000000000000347 25057753

[34] Tevlin R, Borrelli MR, Irizarry D et al (2019) Acellular dermal matrix reduces myofibroblast presence in the breast capsule. Plast Reconstr Surg Glob Open 7:e2213–e2213. 10.1097/GOX.0000000000002213 31333946 10.1097/GOX.0000000000002213 PMC6571298

[35] Moyer HR, Pinell-White X, Losken A (2014) The effect of radiation on acellular dermal matrix and capsule formation in breast reconstruction: clinical outcomes and histologic analysis. Plast Reconstr Surg 133:214–221. 10.1097/01.prs.0000437255.01199.42 24469157 10.1097/01.prs.0000437255.01199.42

[36] Hwang K, Sim HB, Huan F, Kim DJ (2010) Myofibroblasts and capsular tissue tension in breast capsular contracture. Aesthetic Plast Surg 34:716–721. 10.1007/s00266-010-9532-8 20512331 10.1007/s00266-010-9532-8

[37] Brazin J, Malliaris S, Groh B et al (2014) Mast cells in the periprosthetic breast capsule. Aesthetic Plast Surg 38:592–601. 10.1007/s00266-014-0318-2 24811971 10.1007/s00266-014-0318-2

[38] Bassetto F, Scarpa C, Caccialanza E et al (2010) Histological features of periprosthetic mammary capsules: silicone vs. polyurethane. Aesthetic Plast Surg 34:481–485. 10.1007/s00266-010-9483-0 20229111 10.1007/s00266-010-9483-0

[39] Raso DS, Schulte BA (1996) Immunolocalization of keratan sulfate, chondroitin-4-sulfate, and chondroitin-6-sulfate in periprosthetic breast capsules exhibiting synovial metaplasia. Plast Reconstr Surg 98:78–82. 10.1097/00006534-199607000-00012 8657791 10.1097/00006534-199607000-00012

[40] Katzin WE, Centeno JA, Feng LJ et al (2005) Pathology of lymph nodes from patients with breast implants: a histologic and spectroscopic evaluation. Am J Surg Pathol 29:506–511. 10.1097/01.pas.0000155145.60670.e4 15767806 10.1097/01.pas.0000155145.60670.e4

[41] Lesesne CB (1997) Textured surface silicone breast implants: histology in the human. Aesthetic Plast Surg 21:93–96. 10.1007/s002669900091 9143423 10.1007/s002669900091

[42] de Bakker E, Zada L, Schmidt RW et al (2023) Baker-IV capsular contracture is correlated with an increased amount of silicone material: an intra-patient study. Plast Reconstr Surg Publish Ah. 10.1097/PRS.0000000000010359 10.1097/PRS.0000000000010359 PMC10666937 36877628

[43] Larsen A, Bak EEF, Hart LB et al (2024) Silicone leakage from breast implants is determined by silicone cohesiveness: a histological study of 493 patients. Plast Reconstr Surg. 10.1097/PRS.0000000000011395 39591364 10.1097/PRS.0000000000011395

[44] Larsen A, Timmermann AM, Kring M et al (2024) Development and validation of a diagnostic histopathological scoring system for capsular contracture based on 720 breast implant capsules. Aesthet Surg J. 10.1093/asj/sjae050 38429010 10.1093/asj/sjae050

