# Supplementary material for: A Histological Assessment Tool for Breast Implant Capsules Validated in 480 Patients with and Without Capsular Contracture
Source: Aesthetic Plast Surg. 2024 Jun 7;49(2):497–508. doi: 10.1007/s00266-024-04128-5 (PMC11813994; doi:10.1007/s00266-024-04128-5)
Supplement: Supplementary file 1 — Electronic Supplementary Material 1. A reference library of histological images illustrating each parameter of the semiquantitative assessment tool with detailed instructions on how to score each parameter. Supplementary file1 (PDF 4214 kb) [file 266_2024_4128_MOESM1_ESM.pdf]

Histological semiquantitative assessment tool for breast implant capsules

Thickness of the collagen layer

Location: The collagen layer constitutes the majority of the capsule and is found situated below the synovial-like metaplasia.
Morphology: A layer of densely packed collagen fibers that varies greatly in thickness, density and can in some cases be seen as separated collagen bundles.
How to score:

- This parameter focuses on the thickness of the collagen layer and the measure does not include the synovial-like metaplasia, the stromal layer, the adventitia, adipose tissue or muscle tissue.
- Choose the most representative area and avoid measurement of thickness in areas where an increase may potentially be caused by folds in the sample, artefacts or the angle that the section has been cut.

<400 µm	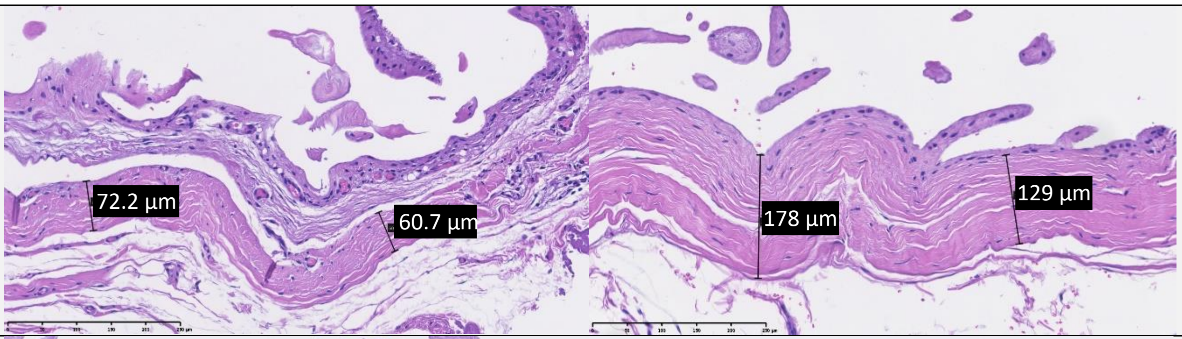
400-600 µm	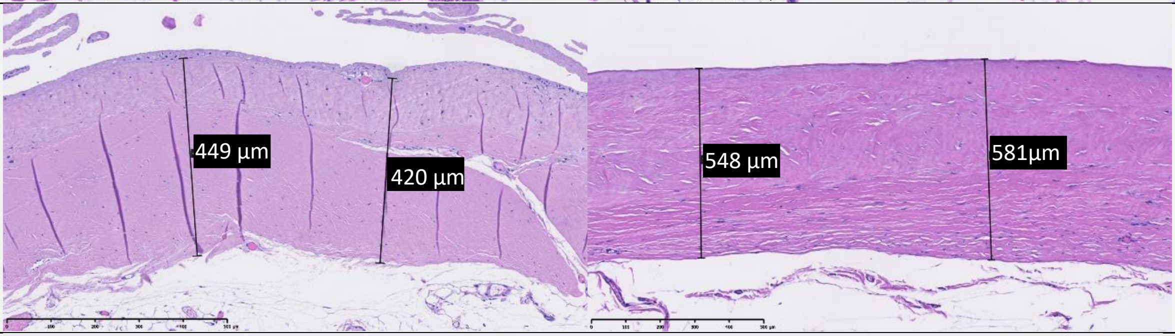
600-800 µm	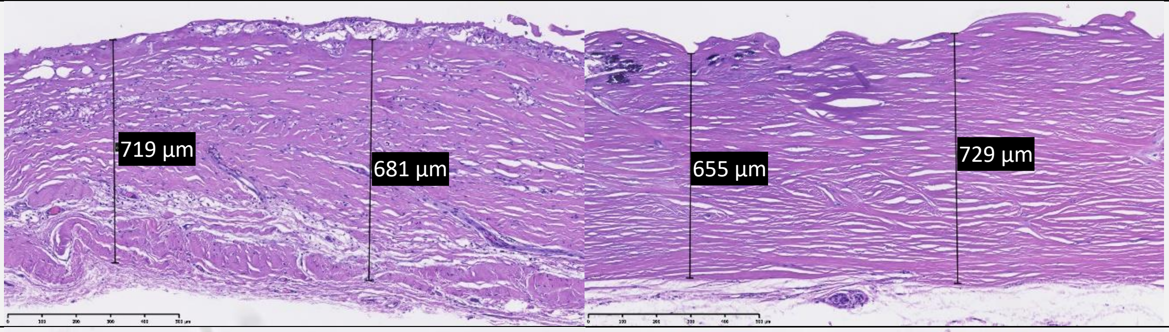
>800 µm	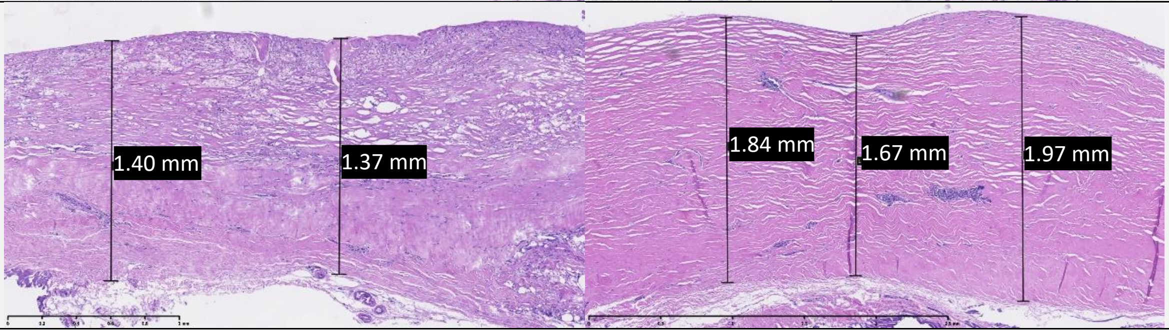

Organization of fibers

Location: The collagen layer.
Morphology: Unorganized fibers are seen as multidirectional bundles of fibers with rounder fibroblast nuclei. Organized fibers are often seen running parallel to the implant surface and fibroblast nuclei are often seen elongated in the direction of traction and parallel to the fibers.
How to score:

- Scoring of the fiber organization may be guided by assessing the direction and shape of the nucleui of the fibroblasts including assessment of the orientation of the nuclei e.g., cross section or longitudinal section.
- Be aware that the way the section is cut may influence how a bundle of collagen fibers that runs parallel to the implant surface may look. If it is cut in cross-section the fibroblasts may seem rounder in shape even if they are elongated in the direction of traction.

Highly disorganized	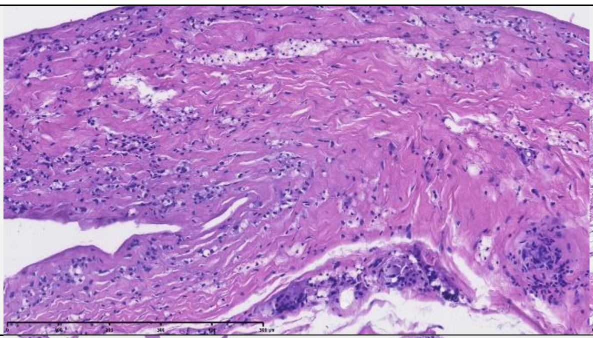 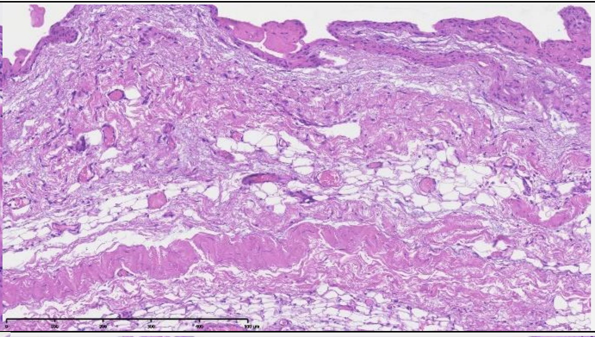
Moderately disorganized	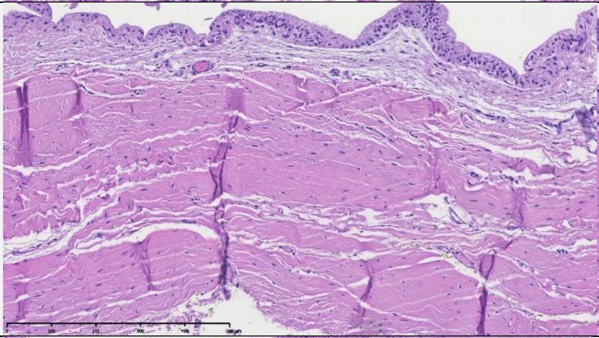 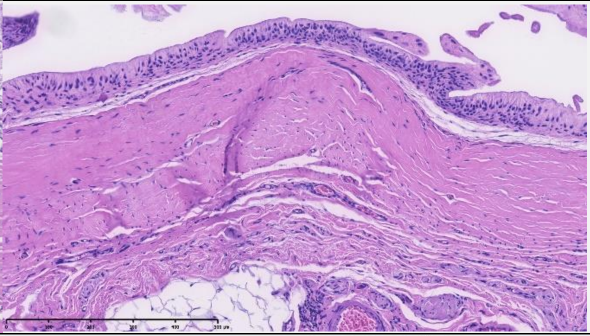
Highly organized	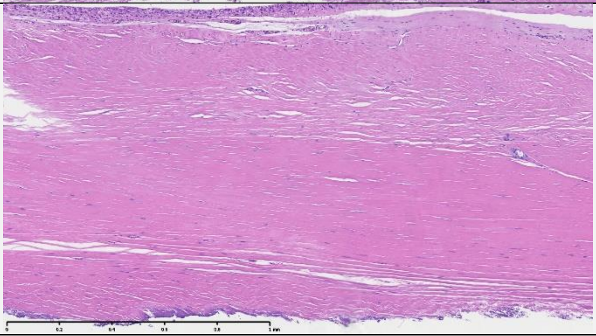 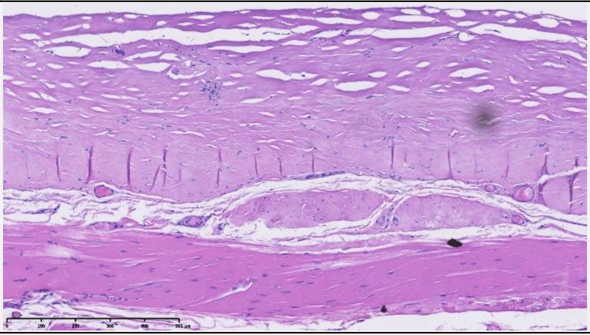

Histological semiquantitative assessment tool for breast implant capsules

Cellularity of in the collagen layer

Location: The collagen layer.
Morphology: Cells are seen as purplish-blue nuclei between collagen fibers in an H&E-stained section. Resident cell populations include fibrocytes, fibroblasts, myofibroblasts and macrophages.
How to score:

- Avoid scoring an area with folds as this may contribute to an appearance of higher cellularity

Few fibroblasts and macrophages	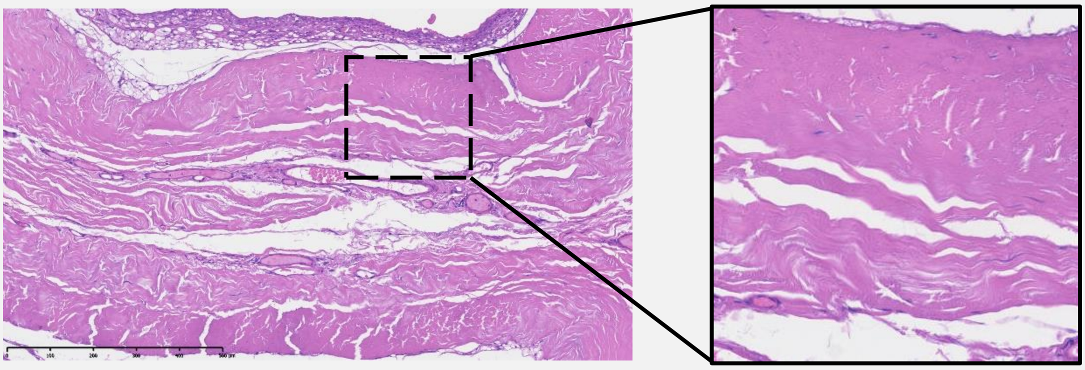
Several fibroblasts and macrophages	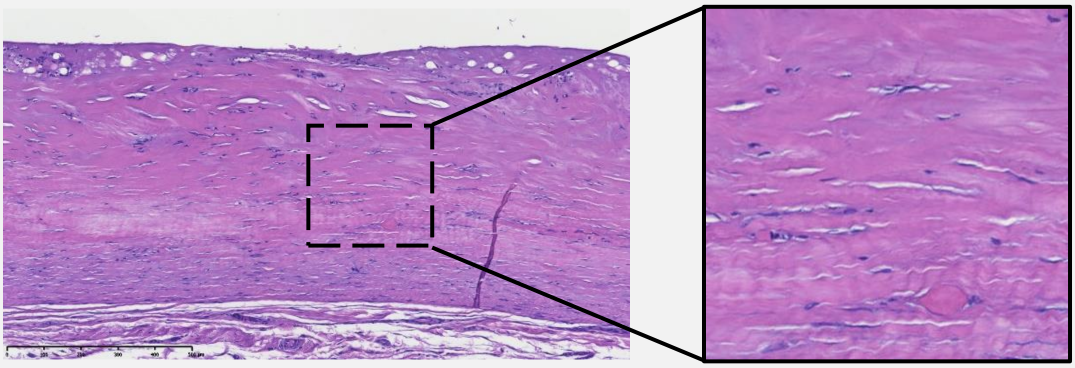
Many fibroblasts and macrophages	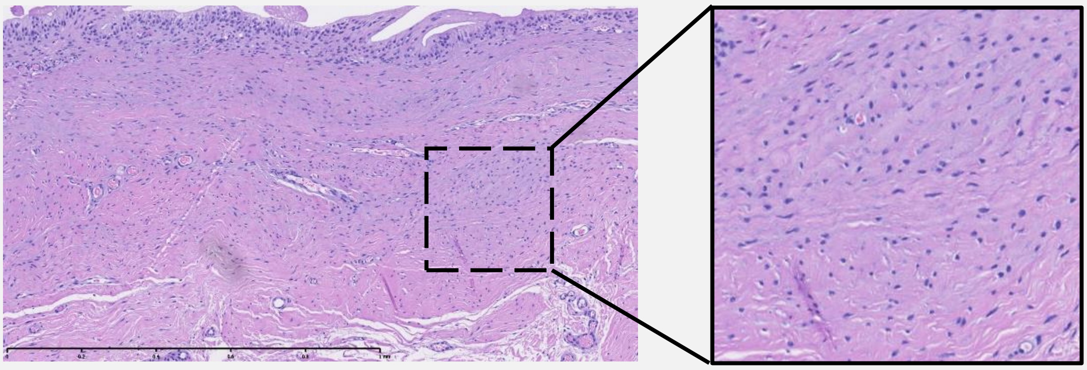

Inflammatory infiltration

Location: Lymphocytes can be found throughout the entire capsule and adventitia.
Morphology: Lymphocytes have small spherical nucleus and abundant dark staining condensed chromatin.
How to score: Lymphocytes can be seen as single induvial cells or in larger aggregations of cells including follicular aggregates.

No or few single inflammatory cells	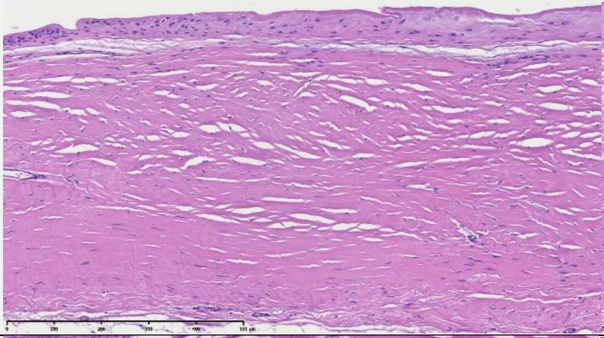	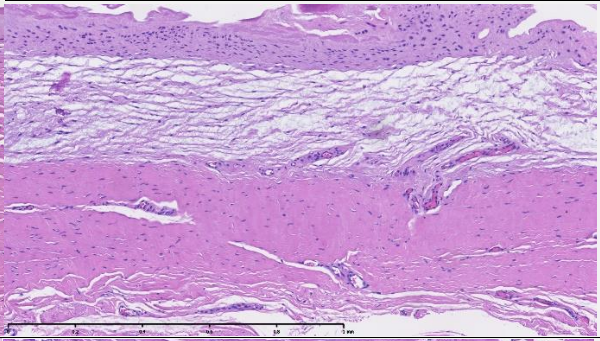
Several single inflammatory cells and few aggregates	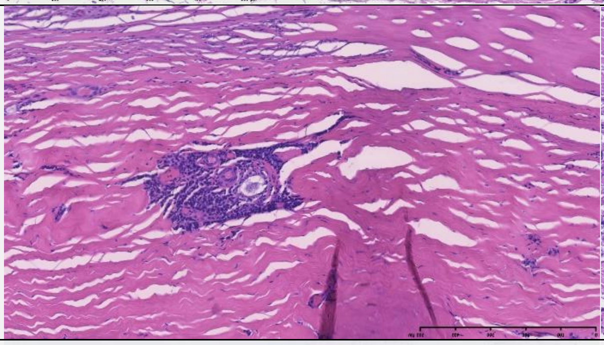	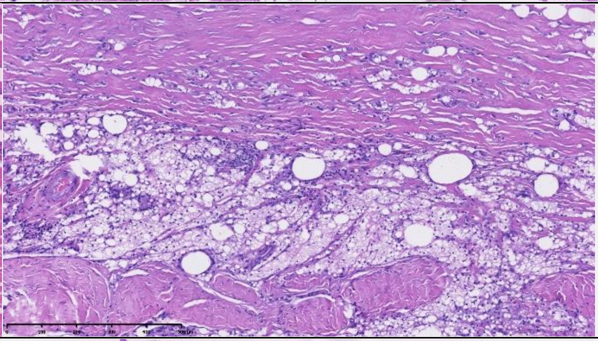
Severe infiltration with several follicular aggregates	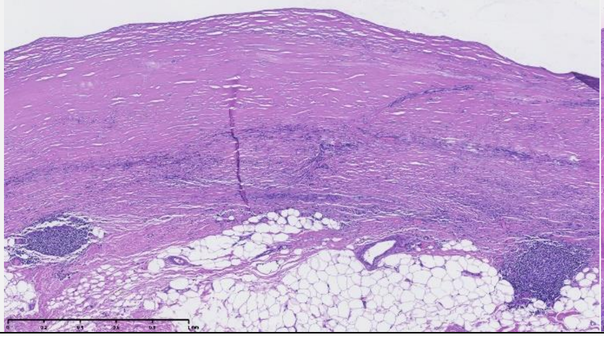	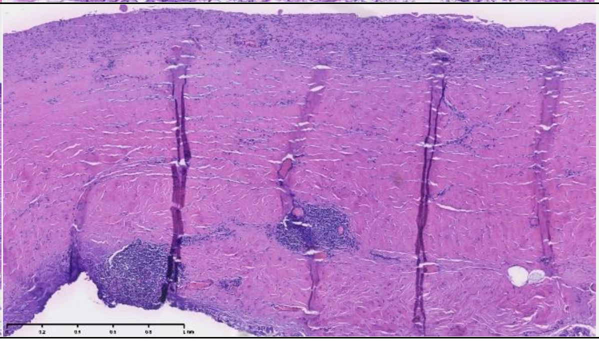

Calcification

Location: Deposition of calcium may be seen throughout the entire capsule.
Morphology: Calcification appears as deep blue purple areas on an H&E-stained section.
How to score:

- Areas of the capsule that have been exposed to heat e.g., from electrosurgical cutting may on an H&E-stained section have a darker appearance. This may potentially be misinterpreted as calcification. We have included examples of this under the category “calcification absent”
- Make sure that your sample is not treated with formic acid in the tissue preparation as this will decalcify your sample and impair the analysis.

Calcification absent	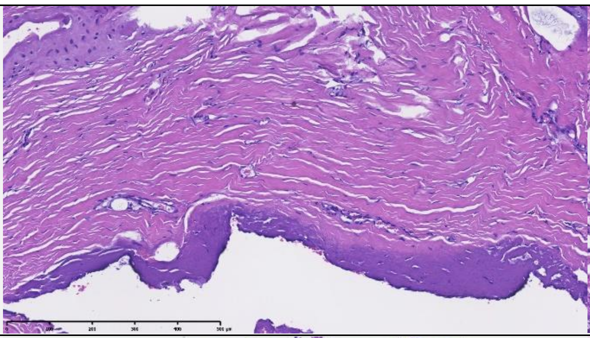	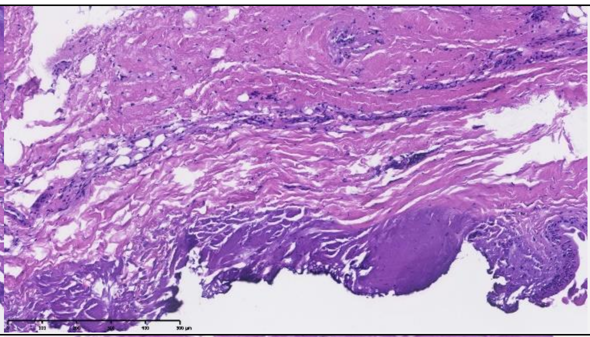
Calcification present	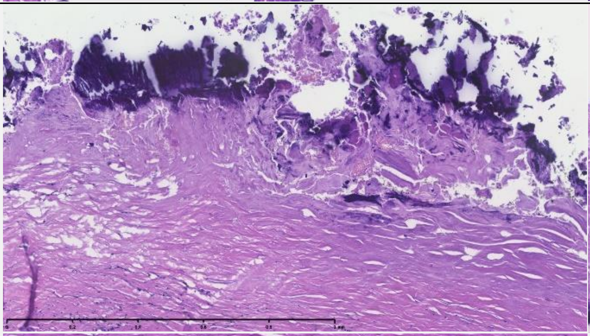	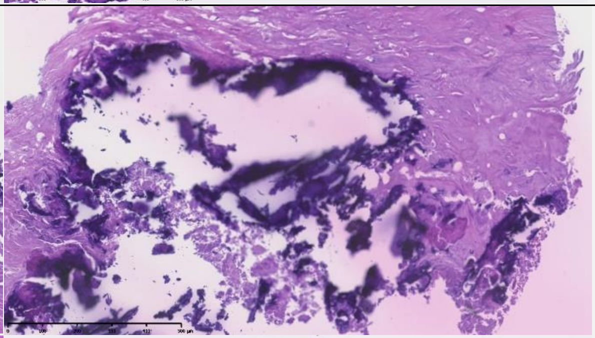
	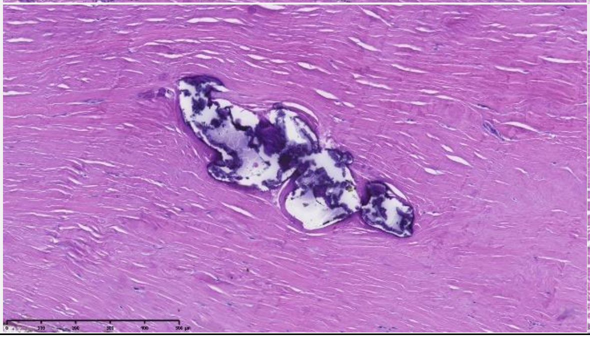	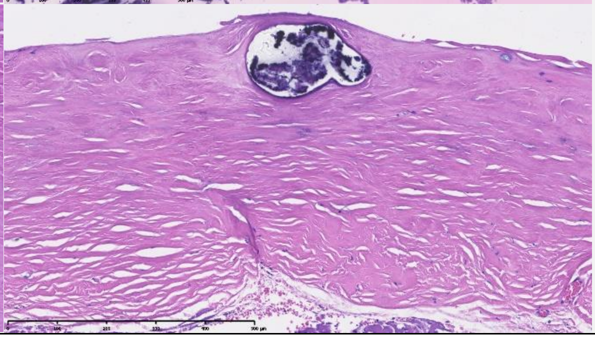

Vascularization

Location: Blood vessels may be seen in the stromal layer and in the collagen layer
Morphology: Blood vessels may be seen with varying diameter and perivascular cells.
How to score: We have provided an example of high vascularization without noticeable inflammation (left) and high vascularization with the presence of inflammatory infiltration (right)

Low vascularization	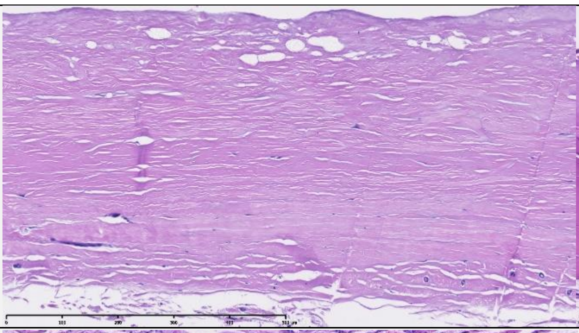 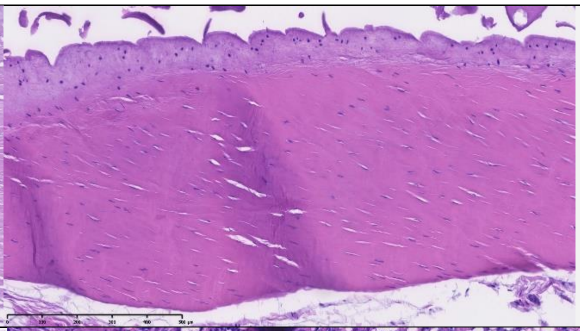
High vascularization	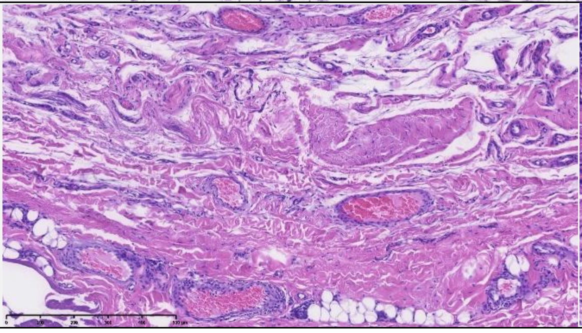 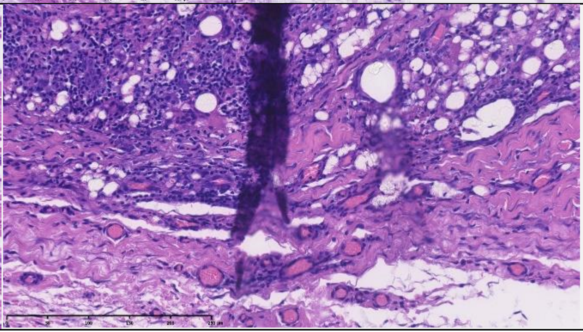

Synovial-like metaplasia

Location: The layer is seen adjacent to the implant surface.
Morphology: The layer consists of macrophage-like and fibroblast-like synovial cells. The cells can be seen as both epithelioid-like and columnar having round to oval nuclei with abundant eosinophilic cytoplasm.
How to score:

- Choose the most representative area of the capsule and score the number of cell layers that is most frequently present.
- Avoid scoring areas where the number of cell layers may be increased due to artefact, folds from sample preparation or from the angle that the section has been cut. Choose an area where the transition from synovial-like metaplasia to underlying tissue is straight without folds.

No synovial-like metaplasia	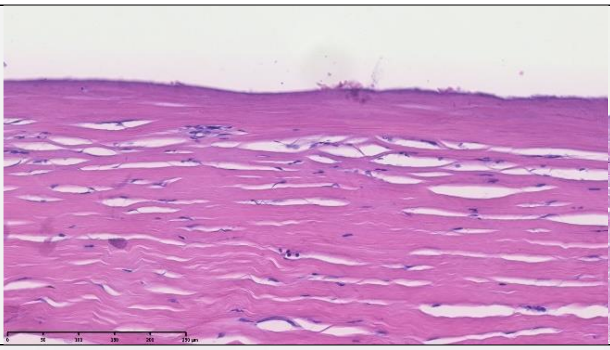	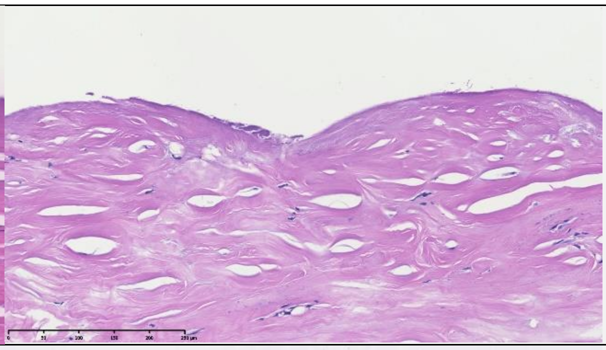
1-2 cell layers	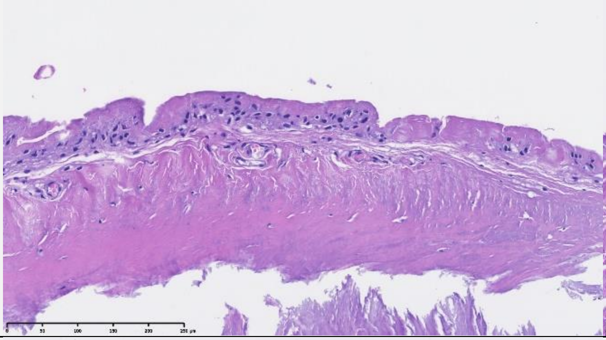	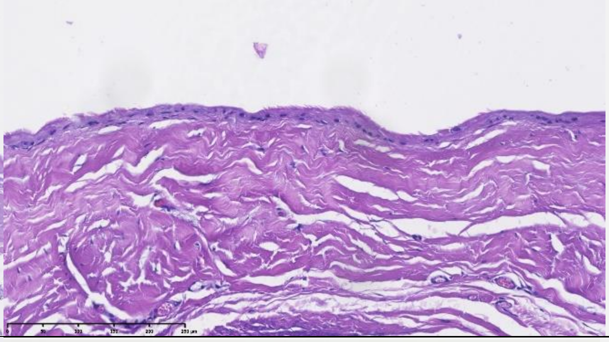
3-4 cell layers	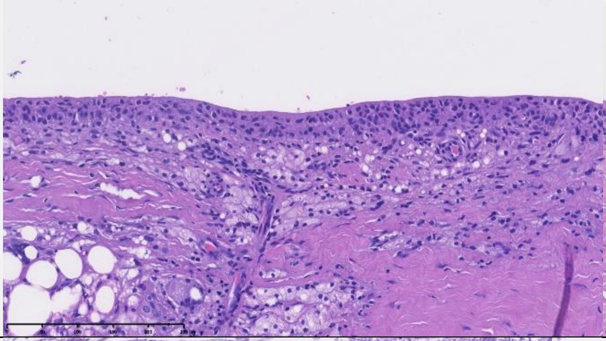	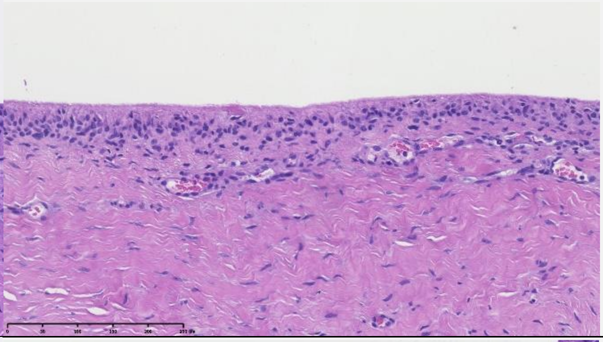
>5 cell layers	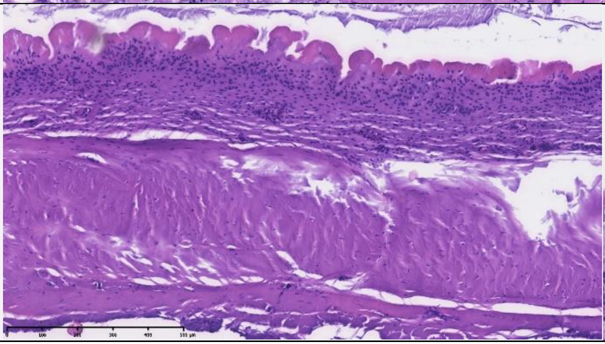	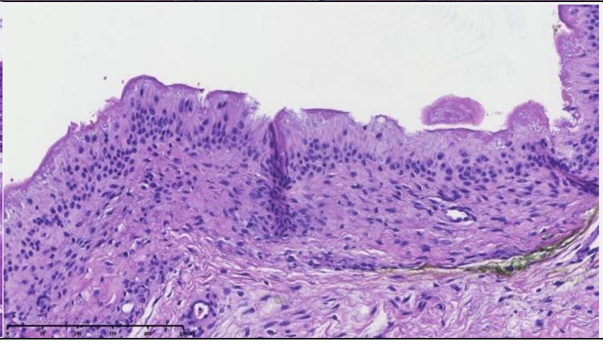

Histological semiquantitative assessment tool for breast implant capsules

Silicone deposition

Location: Silicone can be found throughout the entire capsule including the synovial-like metaplasia.
Morphology: Silicone may be seen as small round to irregular translucent droplets and larger vacuoles of an amorphous refractile material. It may also be located intracellularly when phagocytized by histocytes giving them a foam cell appearance.
How to score:

- Shrinkage artefacts and small vessels without erythrocytes may resemble the appearance of empty silicone vacuoles as silicone is often lost during tissue preparation.
- Synthetic mesh integrated in the capsule is an amorphous foreign material similar to silicone and may be mistaken as silicone droplets.

Absent or nearly absent	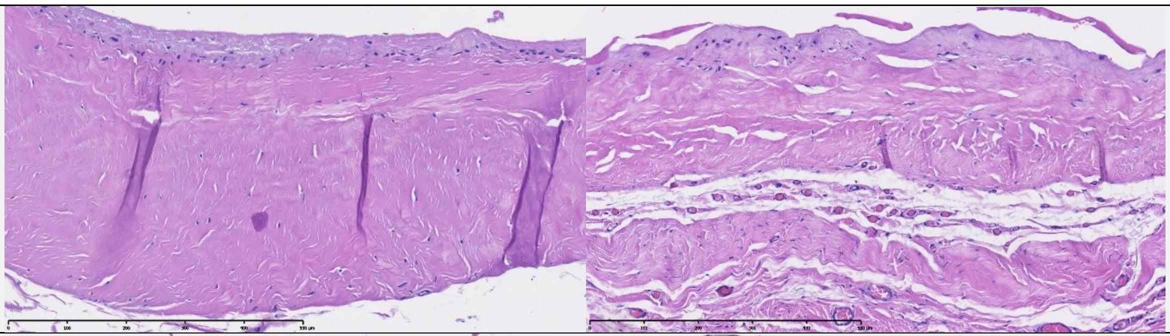
Few silicone vacuoles and/or foam cells	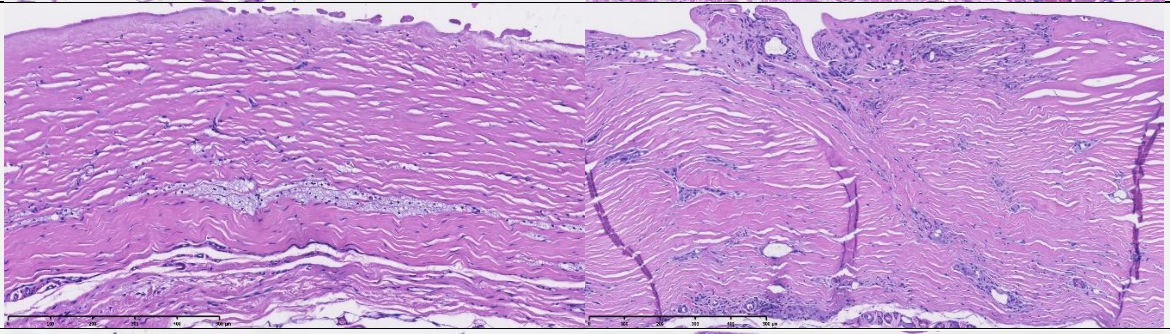
Several silicone vacuoles and/or foam cells	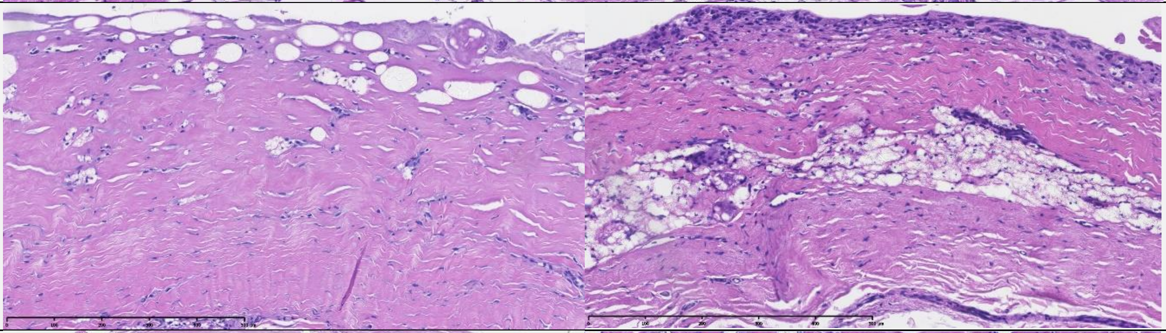
Abundant silicone vacuoles and/or foam cells	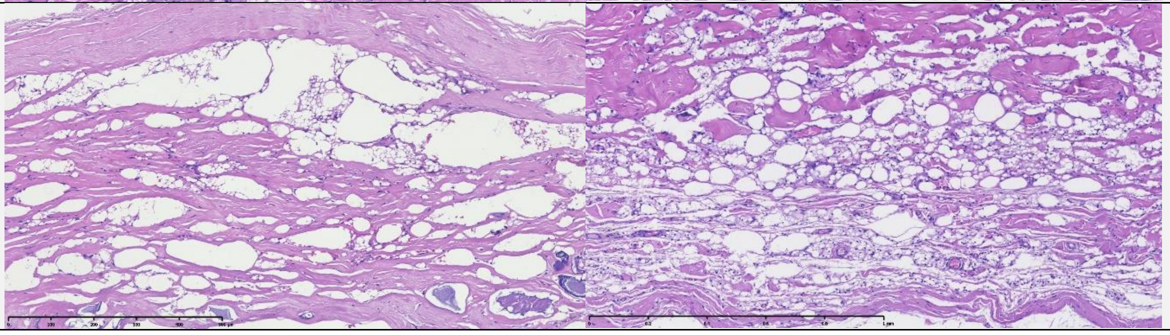

Multinucleated giant cells

Location: Multinucleated giant cells can be seen throughout the entire capsule but are often closely related to the presence of silicone vacuoles or foam cells.
Morphology: Multinucleated giant cells are seen as a fusion of histocytes as a foreign-body response to silicone or as a response to infection and inflammation.
How to score:

- "Few giant cells" if 1-3 multinucleated giant cells are found in the entire samples.
- "Several giant cells" if >3 multinucleated giant cells are found in the entire samples.

Giant cells absent	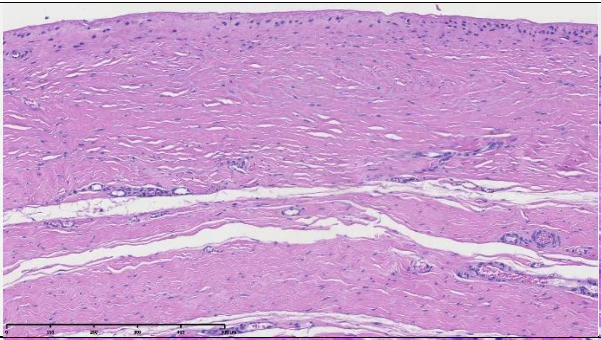	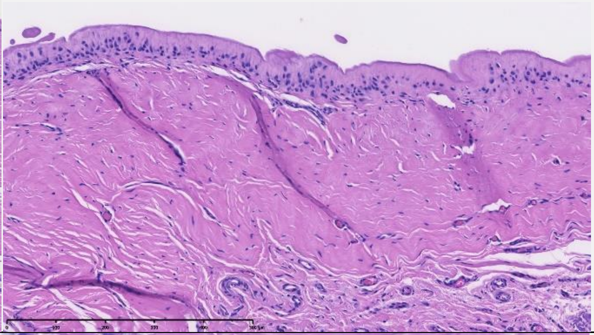
Few giant cells	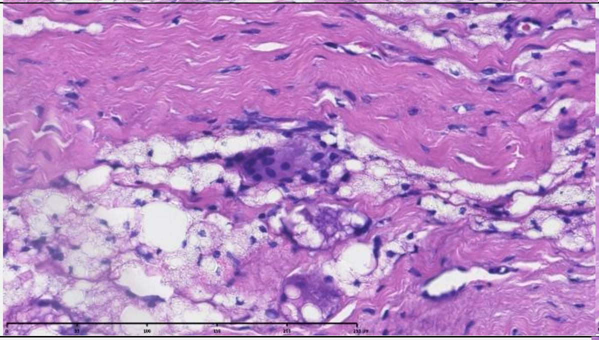	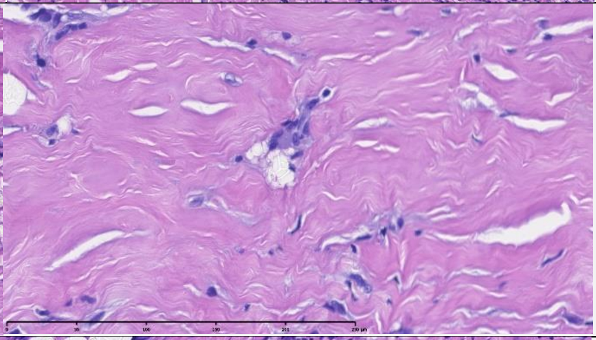
Several giant cells	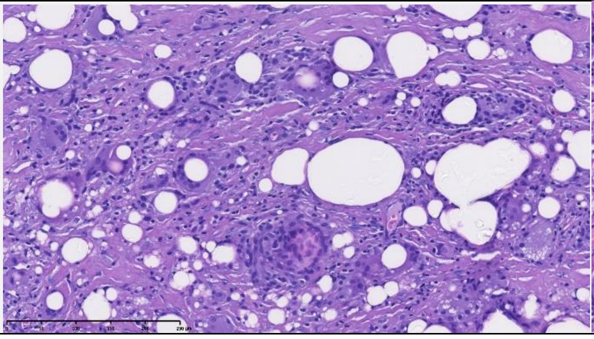	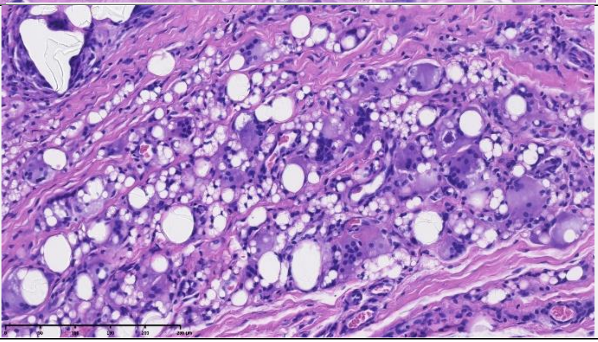

Histological semiquantitative assessment tool for breast implant capsules

Activity in stromal layer

Location: Stroma is often present between the synovial-like metaplasia and the layer of dense collagen fibers but can also be seen between bundles of densely packed collagen fibers.
Morphology: Stroma supports the fibrous capsule and is made up of loose connective tissue and blood vessels.
How to score:

- The presence of the stromal layer is easiest to identify if the layer is thick.
- A thin layer of stroma or severe silicone infiltration may impair the scoring.
- Be aware that artefacts in the tissue can be misinterpreted as a stromal layer.

Absent stromal layer	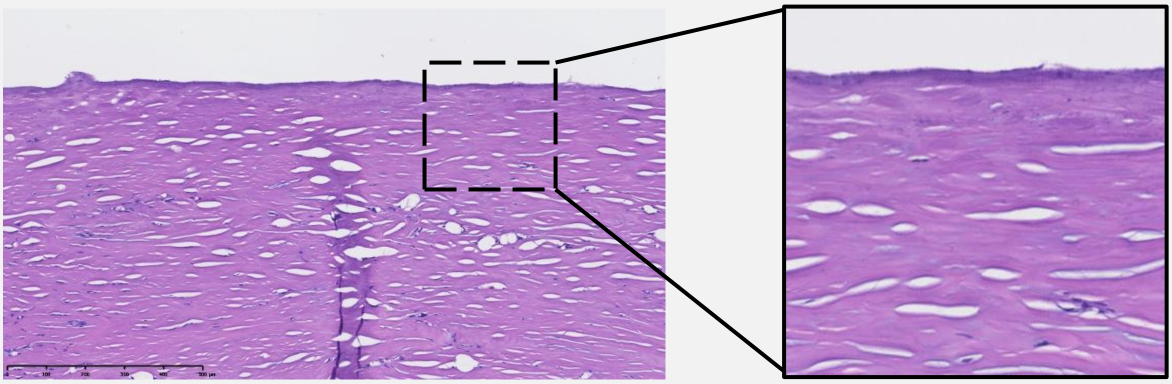
Present with low cellularity	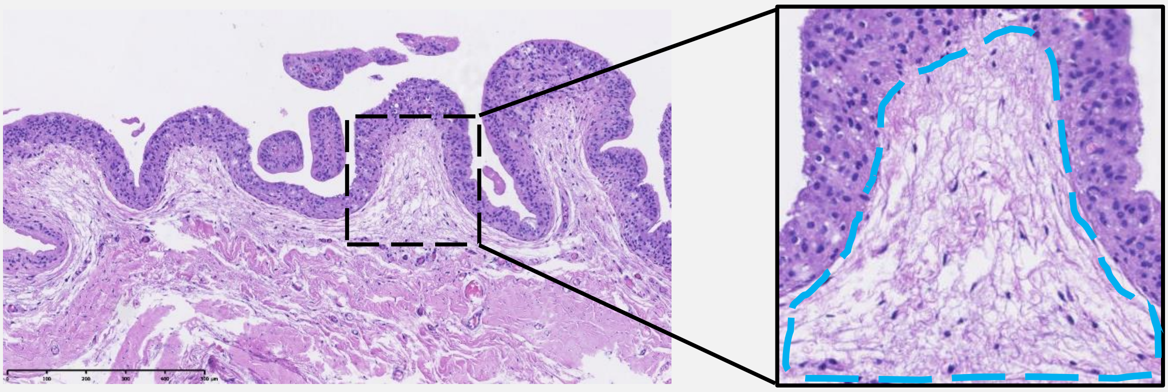
Present with high cellularity	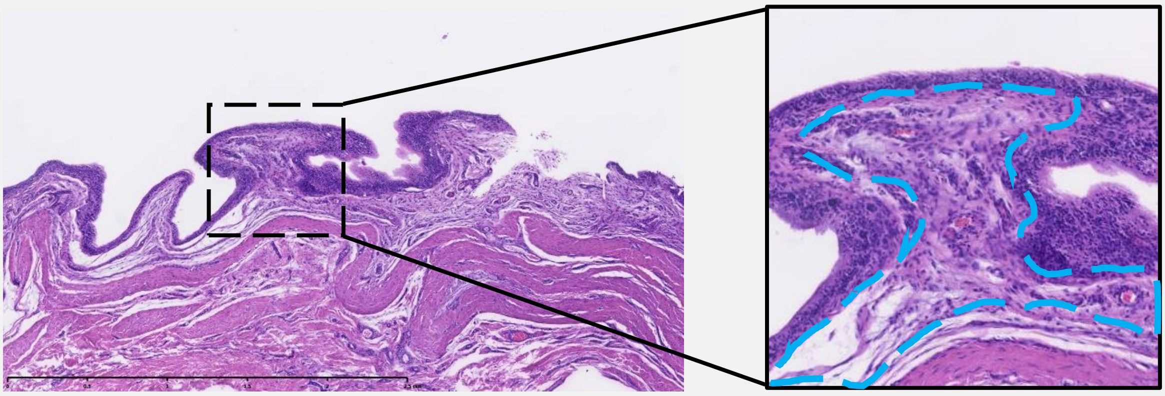

Examples of excluded biopsies

Low quality biopsies excluded from analysis	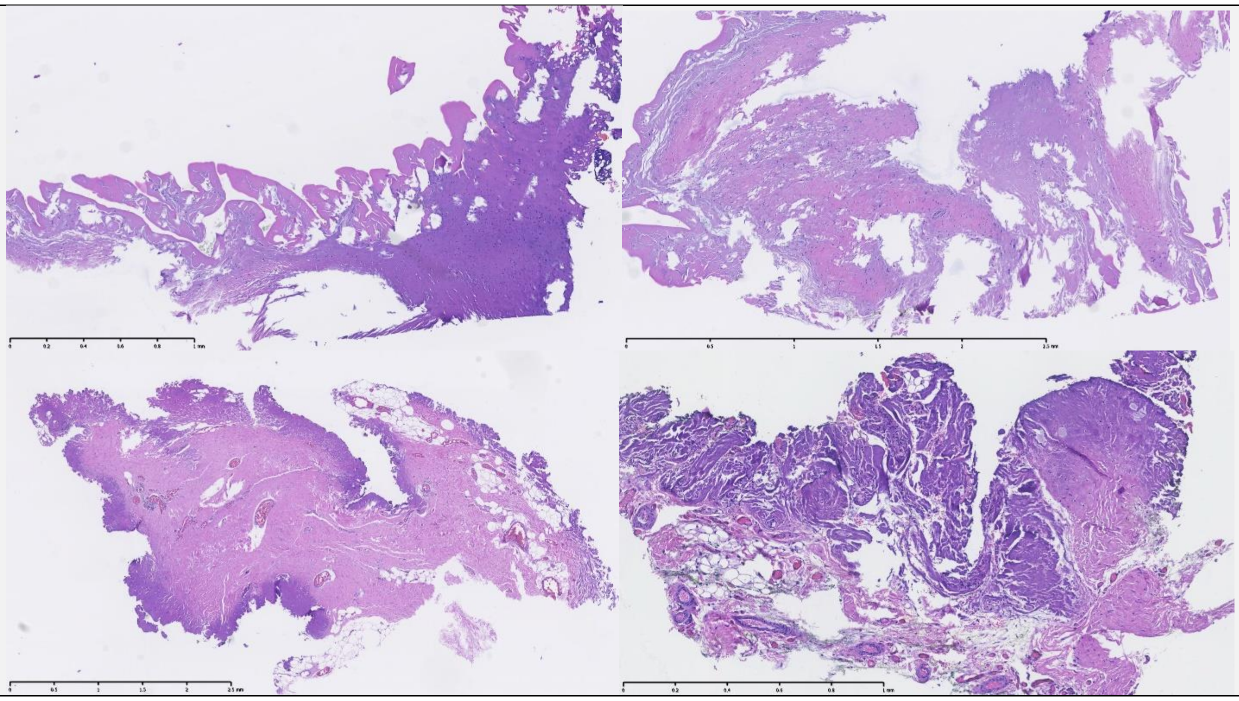
